# Anisotropic Response of Defect Bound States to the Magnetic Field in Epitaxial FeSn Films

**DOI:** 10.1021/acs.nanolett.4c05337

**Published:** 2025-03-13

**Authors:** Huimin Zhang, Zhengfei Wang, Michael Weinert, Lian Li

**Affiliations:** † Department of Physics and Astronomy, West Virginia University, Morgantown, West Virginia 26506, United States; ‡ China Key Laboratory of Materials Modification by Laser, Ion and Electron Beams, Dalian University of Technology, Ministry of Education, Dalian, 116024, China; § Department of Physics, Dalian University of Technology, Ministry of Education, Dalian 116024, China; ∥ Hefei National Research Center for Physical Sciences at the Microscale, CAS Key Laboratory of Strongly-Coupled Quantum Matter Physics, Department of Physics, Hefei National Laboratory, University of Science and Technology of China, Hefei, Anhui 230026, China; ⊥ Department of Physics, University of Wisconsin, Milwaukee, Wisconsin 53201, United States

**Keywords:** Kagome antiferromagnet FeSn, defect bound states, anomalous Zeeman shift, electronic nematicity, STM, MBE

## Abstract

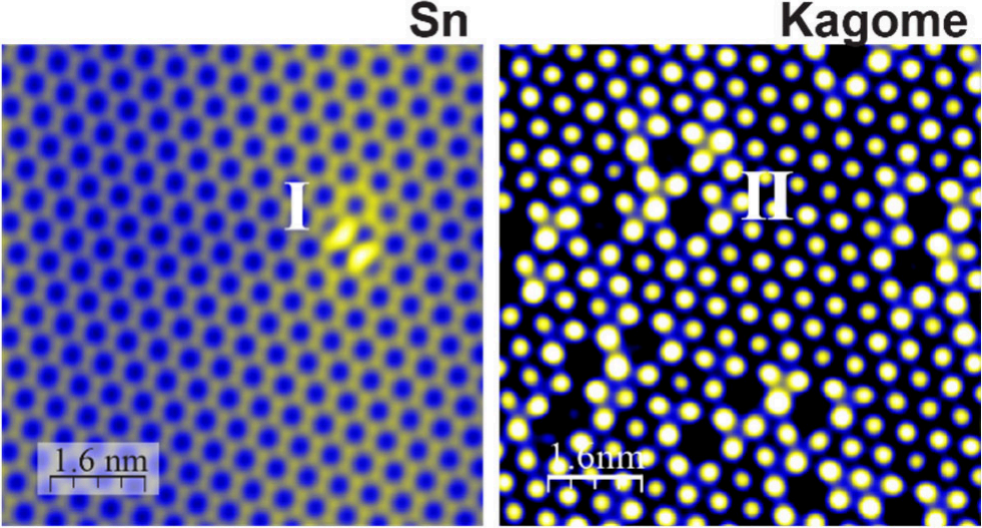

Crystal defects, whether intrinsic or engineered, drive many fundamental phenomena and novel functionalities of quantum materials. Here, we report symmetry-breaking phenomena induced by Sn vacancy defects on the surface of epitaxial Kagome antiferromagnetic FeSn films using low-temperature scanning tunneling microscopy and spectroscopy. Near the single Sn vacancy, anisotropic quasiparticle interference patterns are observed in the differential conductance d*I*/d*V* maps, breaking the 6-fold rotational symmetry of the Kagome layer. Furthermore, the Sn vacancy defects induce bound states that exhibit anomalous Zeeman shift under an out-of-plane magnetic field, where the energy of the bound states moves linearly toward higher energy independent of the direction of the magnetic field. Under an in-plane magnetic field, the shift of the bound state energy also shows a 2-fold oscillating behavior as a function of the azimuth angle. These findings demonstrate defect-enabled new functionalities in Kagome antiferromagnets for potential applications in nanoscale spintronic devices.

Defects in crystals are ubiquitous, whether they form during crystal growth or are induced by postgrowth processing. These defects can significantly modify the physical and electronic properties of quantum materials, enabling new and tunable functionalities. For example, the nitrogen vacancy (NV) color centers in diamond crystals have been identified as a promising candidate for quantum computing qubits due to their optically addressable electron spin states and long coherence time.^1−3^ In high-temperature superconductors, nonmagnetic impurities can break up Cooper pairs and introduce bound states within the superconducting gap, which provides insights into the nature of unconventional superconductivity.^4−7^ The phase-referenced quasiparticle interference (QPI) technique^7−11^ was developed to determine the unconventional pairing symmetry based on the scattering by these nonmagnetic defects.

The recent studies of defect-induced bound states in Kagome materials have also provided significant insight into the interplay of topology, charge-ordered phases, and magnetism. The Kagome lattice, which forms the building block of these materials, is a two-dimensional (2D) network of corner-sharing triangles and hexagons (Figure 1a). This unique structure gives rise to linearly dispersing Dirac cones at the Brillouin zone (BZ) corner K point, Von Hove singularities at the M point, and a flat band across the entire BZ.^12^ These electronic band features have already been confirmed by angle-resolved photoemission spectroscopy (ARPES) in binary metallic Kagome magnets T_*m*_X_*n*_ (T: 3d transition metals, X: Sn, Ge, *m*:*n* = 3:1, 3:2, 1:1)^13−16^ and ternary ferromagnetic YMn_6_Sn_6_.^17^ Fascinating phenomena have also been reported, such as giant spin–orbit tunability of the Dirac mass and electronic nematicity in Fe_3_Sn_2_,^18,19^ magnetic Weyl semimetal state and negative flat band magnetism in Co_3_Sn_2_S_2_,^20−22^ topological Chern magnet in the quantum limit in TbMn_6_Sn_6_,^23^ and orbital Zeeman effect in TbV_6_Sn_6_.^24^ Furthermore, nonmagnetic substitutional indium impurity in Co_3_Sn_2_S_2_ was shown to introduce spin-polarized bound states consistent with a negative orbital magnetization.^25^ The sulfur vacancy in Co_3_Sn_2_S_2_ also exhibits negative orbital magnetization, which was attributed to spin–orbit polarons.^26,27^

**Figure 1:**
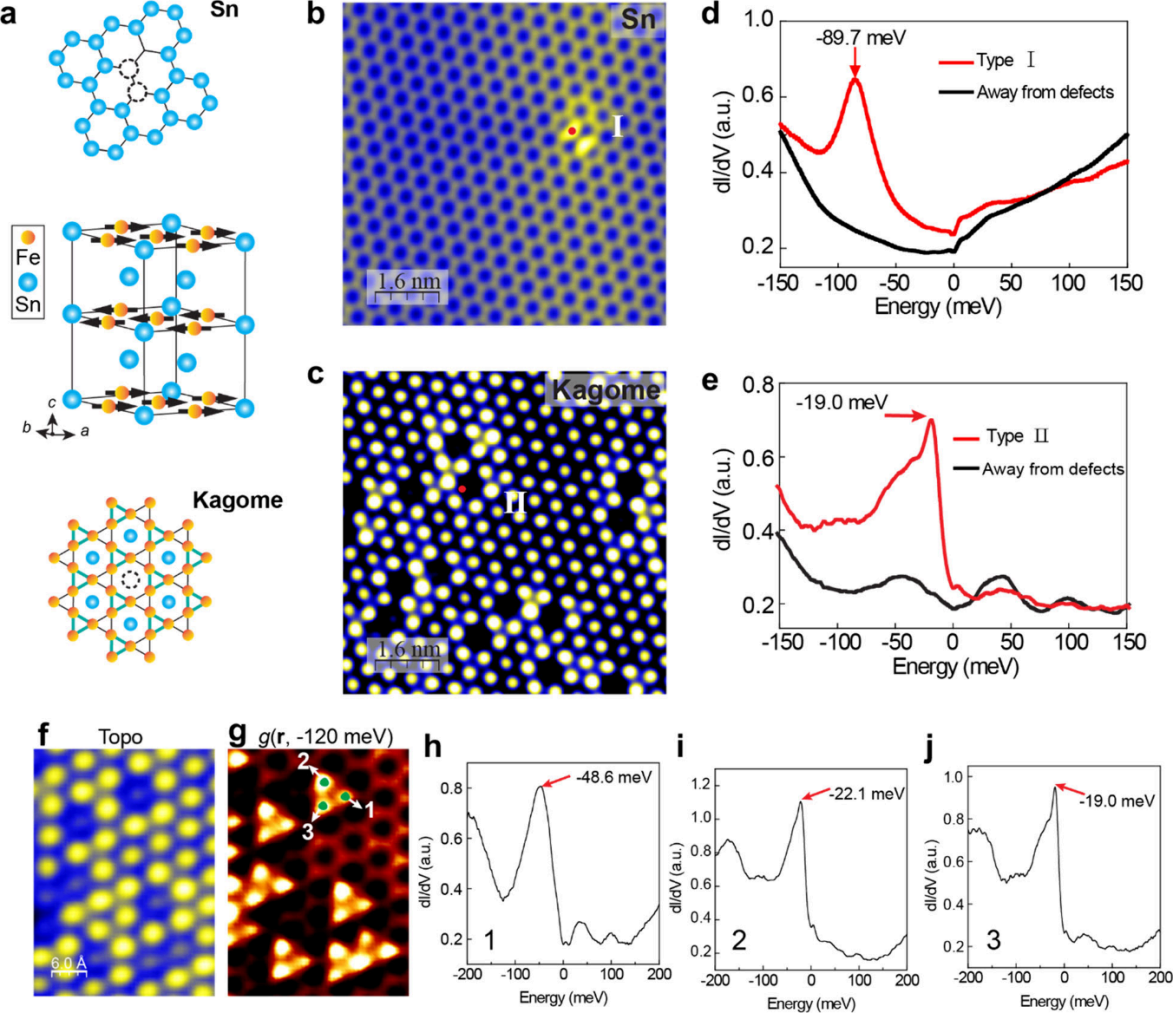
STM imaging and spectroscopy of MBE-grown FeSn films on the SrTiO_**3**_(111) substrate. **a**, Ball-and-stick model of FeSn consisting of vertical stacks of stanene (Sn) and Kagome layers. **b**, **c**, Topographic STM images of two terminations: the Sn layer (**b**) and the Kagome layer (**c**). Set point: *V* = 0.5 V, *I* = 3.0 nA (**b**) and *V* = −0.5 V, *I* = 3.0 nA (**c**). **d**, **e**, Differential conductance d*I*/d*V* spectra taken on type I and type II defects (red curve) compared to that taken away from the defects (black curve). **f**, Topographic STM image of Sn vacancies on the Kagome layer. Set point: *V* = 0.2 V, *I* = 3.0 nA. **g**, d*I*/d*V* map taken at −120 meV in the same field of view as (**f**). Set point: *V* = 0.2 V, *I* = 3.0 nA, *V*_mod_ = 3.0 meV. **h–j**, d*I*/d*V* spectra taken at the three sites marked in (**g**) revealing the bound states.

For antiferromagnetic (AFM) Kagome materials, studies have focused on FeGe, Mn_3_Sn, and FeSn. In addition to the *c*-axis collinear AFM ordering below *T*_N_ ≈ 410 K and a double-cone (canted) AFM structure below *T*_Canting_ ≈ 60 K,^28,29^ the FeGe surface exhibits edge states and a (2 × 2) charge order,^30^ which couples strongly to magnetism based on ARPES and neutron scattering studies.^31,32^ On the other hand, Mn_3_Sn shows an in-plane noncollinear AFM order,^33,34^ leading to large anomalous Hall,^35^ anomalous Nernst,^36^ and magneto-optical Kerr^37^ effects. Remarkably, all of these effects are observed at a moderate magnetic field, making it appealing to control the topological electronic states for AFM-based spintronics. For FeSn, which consists of alternatively stacked planes of 2D Fe_3_Sn Kagome layer and Sn_2_ stanene layer,^38−40^ Fe atoms within each Kagome layer exhibit in-plane ferromagnetic order,^38,39^ while neighboring Kagome layers are coupled antiferromagnetically along the *c*-axis with a Néel temperature *T*_N_ ≈ 366 K. For cleaved bulk materials, earlier ARPES studies have revealed linearly dispersed Dirac crossings at −0.43 and −0.31 eV at K point and flat bands at −0.23 eV.^14^ Recently, we have reported symmetry-breaking electronic nematic order tunable by an applied magnetic field,^41^ and strain-induced giant periodic pseudomagnetic fields greater than 1000 T in FeSn epitaxial films.^42^

In this study, we report defect-induced symmetry-breaking phenomena on the surface of epitaxial Kagome antiferromagnet FeSn films using low-temperature scanning tunneling microscopy and spectroscopy (STM/S). Near single Sn vacancy defect on the K-terminated FeSn films, we observe anisotropic QPI patterns in conductance d*I*/d*V* maps, which break the 6-fold rotational symmetry of the Kagome layer. Furthermore, Sn vacancy defects that induced bound states exhibit anomalous Zeeman shifts under an out-of-plane magnetic field, where the energy of the bound states increases linearly with the magnetic field strength, regardless of the field direction. When the magnetic field is applied in-plane, the shift of the bound state energy also shows a 2-fold oscillating behavior as a function of the azimuth angle, demonstrating the potential for field-controlled anisotropic transport for nanoscale spintronic devices.

## Point Defects in Epitaxial Kagome Antiferromagnet FeSn Films

We prepare FeSn films by molecular beam epitaxy (MBE) (see Methods) and confirm their crystal structure by X-ray diffraction measurements.^41^ The epitaxial FeSn/STO(111) films exhibit island growth with two types of terminations (Figure 1a and Figure S1): one with a honeycomb lattice as shown in Figure 1b and the second with a close-packed lattice as shown in Figure 1c, assigned to the Sn and Kagome layers, respectively. Point defects are commonly seen on the Sn layer, which typically shows an enhanced local density of states (LDOS) with a 2-fold symmetry, labeled as type I (Figures 1b and S3). On the other hand, defects on the Kagome layer are often associated with a suppressed density of states, labeled as type II (Figures 1c and S4). To determine the nature of these defects, we carried out first-principles density functional theory (DFT) calculations. The simulated LDOS for both layers with Sn vacancy defects agrees well with the experimental STM images (Figure S5 and Supplementary Note 1). Thus, these type I and II defects are attributed to Sn divacancy on the Sn layer and single vacancy on the Kagome layer, respectively. In addition to the type I Sn divacancy defect discussed above, we observed another type of defect on the Sn termination (Figure S2), likely a substitutional Sn defect, which induces bound states at −61.2 meV. The nature of the substitutional impurity is unknown. Therefore, we mainly focus on type I and II defects in this study.

## Symmetry-Breaking QPI Pattern near Sn Vacancy Defects

d*I*/d*V* tunneling spectroscopy further reveals the differences between the two types of defects (Figures 1d,e). Specifically, type I defects show a bound state at −89.7 meV in the d*I*/d*V* spectrum (red curve in Figure 1d), and type II defects display bound state at −19.0 meV (red curve in Figure 1e). We note that while the bound state energy for the Sn divacancy defect in the Sn layer is fixed, for the Sn single vacancy in the Kagome layer, the energy is spatially dependent as shown in Figures 1f,g and Figure S6, likely due to coupling with neighboring defects, similar to earlier studies.^27^ Both Sn vacancy defects lead to anisotropic QPI patterns nearby in differential conductance d*I*/d*V* maps that represent the densities of states. For Sn divacancy on the Sn layer, the 2-fold symmetrical bound states seen in the topographic images (Figure S3) and d*I*/d*V* maps (Figure S7) are consistent with the divacancy character of the defect.

**Figure 2:**
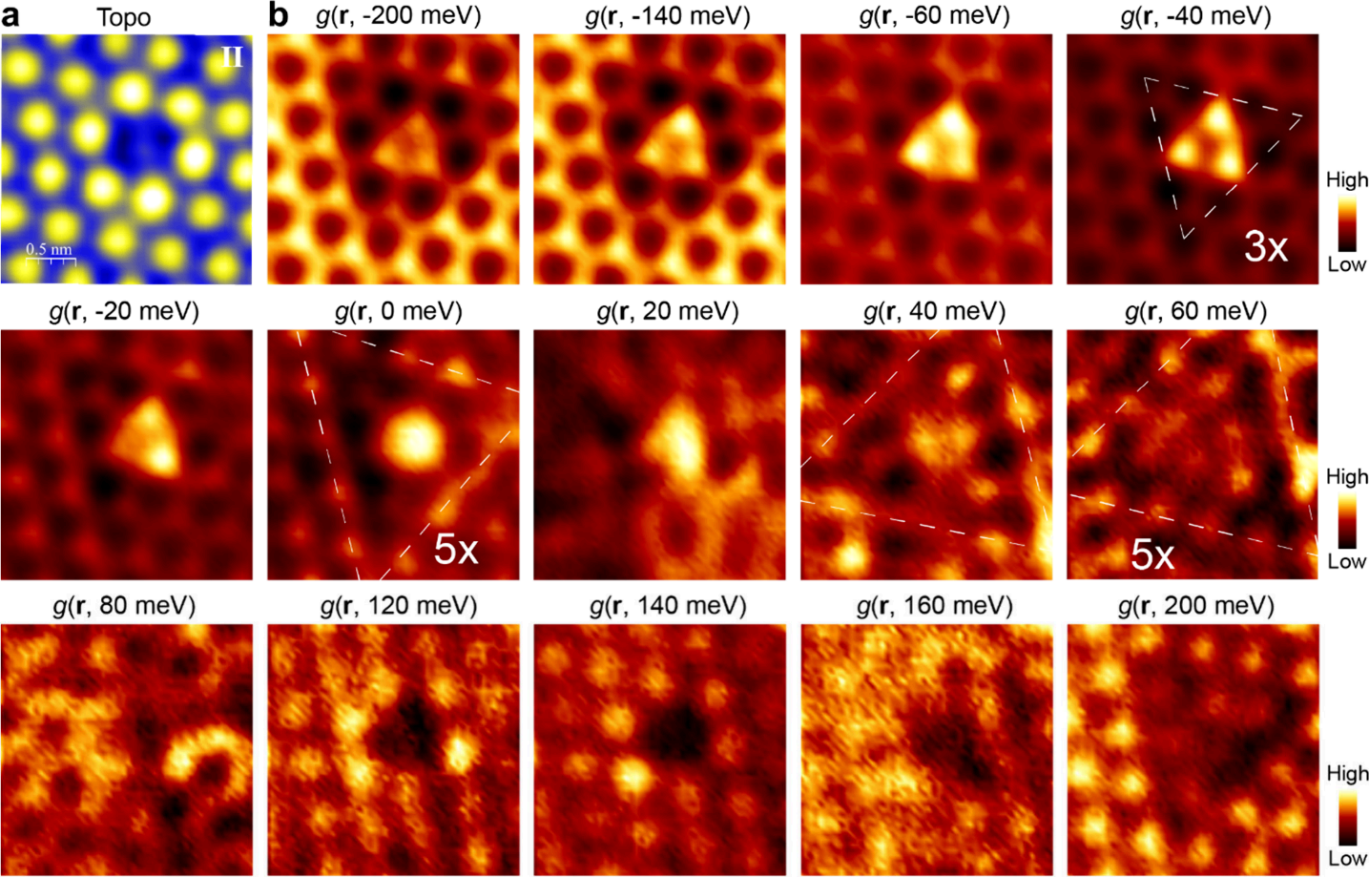
Anisotropic QPI near a Sn vacancy on the Kagome layer in FeSn/STO(111) films. **a**, Topographic STM image of the Sn vacancy (type II defect) on the Kagome layer, set point: *V* = −0.5 V, *I* = 3.0 nA. **b**, d*I*/d*V* maps around the defect, set point: *V* = 0.6 V, *I* = 5.0 nA, *V*_mod_ = 6 mV.

In contrast, for the Sn vacancy on the Kagome layer, the spatial distribution of the bound state displays a complex energy dependence: a trimer within the energy range from −140 to −40 meV and a dimer at −20 meV (Figure 2b). Interestingly, the bound states become featureless from the Fermi level to 20 meV, and a contrast reversal from bright to dark occurs at ∼120 meV (more details in Figure S8). In addition, the defect induces a QPI pattern as a triangular-shaped depression in differential conductance maps (cf. *g*(**r**, −140 meV)). The contrast of the depression is also energy dependent: it is the most pronounced in the energy range between −200 and −100 meV and almost invisible at 20 meV. The triangular pattern also flips from pointing down at *E*_F_ to pointing up at 40 meV, as outlined by the dotted triangles. This breaking of the 6-fold crystal symmetry of the Kagome layer is indicative of nematicity. Similar symmetry-reducing QPI patterns observed near a point defect in strongly correlated Sr_3_Ru_2_O_7_ have also been attributed to nematicity, driven by the interaction of magnetism and spin–orbit coupling.^43^ This signature of electronic nematicity^41^ is further supported by the anisotropic response of the bound states to the magnetic field discussed below.

## Anomalous Zeeman Shift under an out-of-Plane Magnetic Field

**Figure 3:**
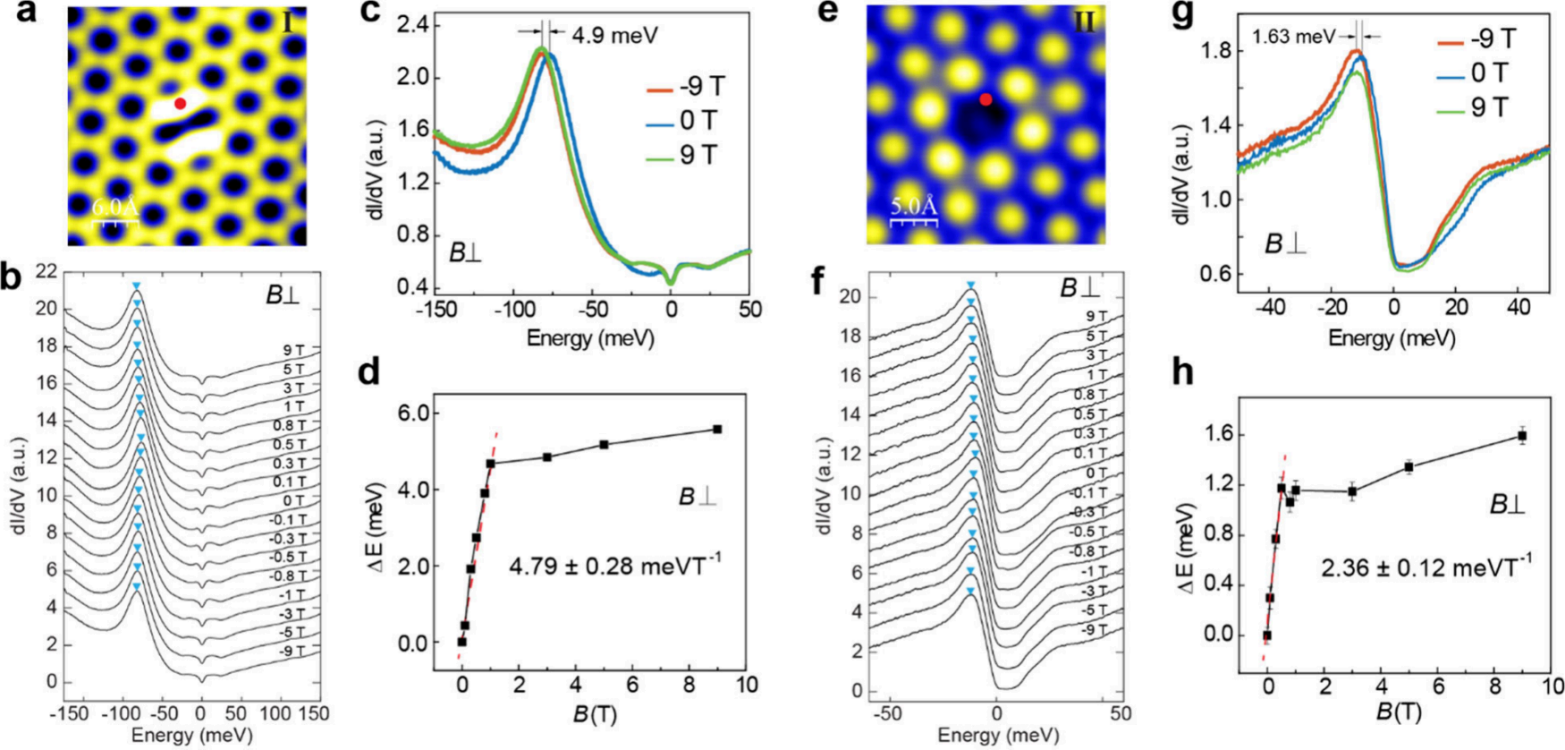
Anomalous Zeeman shift of the defect bound states on the Sn and Kagome layers. **a**, Topographic STM image of Sn divacancy on the Sn layer. Set point: *V* = 0.2 V, *I* = 3.0 nA. **b**, d*I*/d*V* spectra taken under out-of-plane magnetic fields as indicated. The energy positions of the bound states are marked by cyan triangles. **c**, Comparison of the bound states taken at the red dot under 0 T and ±9 T. A relative shift of 4.9 meV is observed. **d**, Linear fitting of the bound states Δ*E* as a function of magnetic field *B*_⊥_ within the range [0, 0.5 T]. The slope is 4.79 ± 0.28 meV·T^–1^, corresponding to an effective *g* factor of 165.2 ± 9.7. **e**, Topographic STM image of a single Sn vacancy on the Kagome layer, set point: *V* = −0.5 V, *I* = 3.0 nA. **f**, d*I*/d*V* spectra of the bound states under various magnetic fields *B*_⊥_. **g**, Comparison of the bound states under 0 T and ±9 T. **h**, Linear fit of the energy shift of the bound states as a function of *B*_⊥_ within the range [0, 0.5 T]. The slope 2.36 ± 0.12 meV·T^–1^ corresponds to an effective *g* factor of 81.4 ± 4.2.

Next, we examined the response of the defect bound states to an out-of-plane magnetic field (*B*_⊥_). For a Sn divacancy (type I), the d*I*/d*V* spectra taken at the same site (red dot in Figure 3a) under various *B*_⊥_ field strengths are shown in Figures 3b,c, and the peak positions are summarized in Figure 3d. The bound states experience a negative energy shift between −0.5 and 0.5 T (here, the positive direction is defined as Δ*E* = (*E*_*B*_ – *E*_*B=0*_) < 0), independent of the magnetic field direction. Specifically, the peak position shifts by Δ*E* = 4.9 meV away from the Fermi level at *B*_⊥_ = −9 or 9 T compared to that under *B*_⊥_ = 0 T (Figure 3c). Before saturation above *B*_⊥_ = 0.5 T, the shift can be well fitted by the linear function Δ*E*_B_ = *g*·Δ*B*, which yields a slope of 4.79 ± 0.28 meV·T^–1^, corresponding to an effective *g* factor of 165.2 ± 9.7 (Figure 3d and Supplementary Note 2). Similar behavior is observed for the single Sn vacancy defect on the Kagome layer (type II) but with a relatively smaller slope of 2.36 ± 0.12 meV·T^–1^, corresponding to an effective *g* factor of 81.4 ± 4.2 (Figures 3e–h). The anomalous Zeeman shift observed here is comparable to that reported earlier in Kagome magnets Co_3_Sn_2_S_2_,^22,25,26^ Fe_3_Sn_2_,^18^ and TbV_6_Sn_6_.^24^ Nevertheless, they are much larger than these in Co_3_Sn_2_S_2_ (0.075 meV·T^–1^, 0.174 meV·T^–1^)^22,25,26^ and smaller than those in Fe_3_Sn_2_ (12 meV·T^–1^),^18^ and TbV_6_Sn_6_ (11.20 meV·T^–1^).

For the normal Zeeman effect, Δ*E* = −μ·*B*, where Δ*E* is the energy shift, μ the magnetic moment, and *B* the applied magnetic field. The energy would decrease if the magnetic moment μ is parallel to the applied magnetic field *B* but would increase if antiparallel. However, when the energy shift Δ*E* is independent of the magnetic field direction, the so-called anomalous Zeeman shift Δ*E* < 0 indicates that the net magnetization of the bound states is always parallel to the direction of the magnetic field, suggesting strong contributions from the orbital moment (Figure S9). Previously, a positive energy shift (Δ*E* > 0) has been observed in magnetic Weyl semimetal Co_3_Sn_2_S_2_, either for the flat band feature^22^ or for the defect-induced bound states (indium-doped or sulfur vacancy).^25,26^ In contrast, a negative energy shift (Δ*E* < 0) was reported for the flat band of ferromagnetic Kagome metal Fe_3_Sn_2_.^22^ The anomalous Zeeman shift also saturates at *B* = 1 T in FeSn (this work) or Fe_3_Sn_2_^18^ and TbV_6_Sn_6_,^24^ but never saturates, even at *B* = 8 T, in Co_3_Sn_2_S_2_.^22,25,26^

## Oscillating Behavior under in-Plane Magnetic Fields

**Figure 4:**
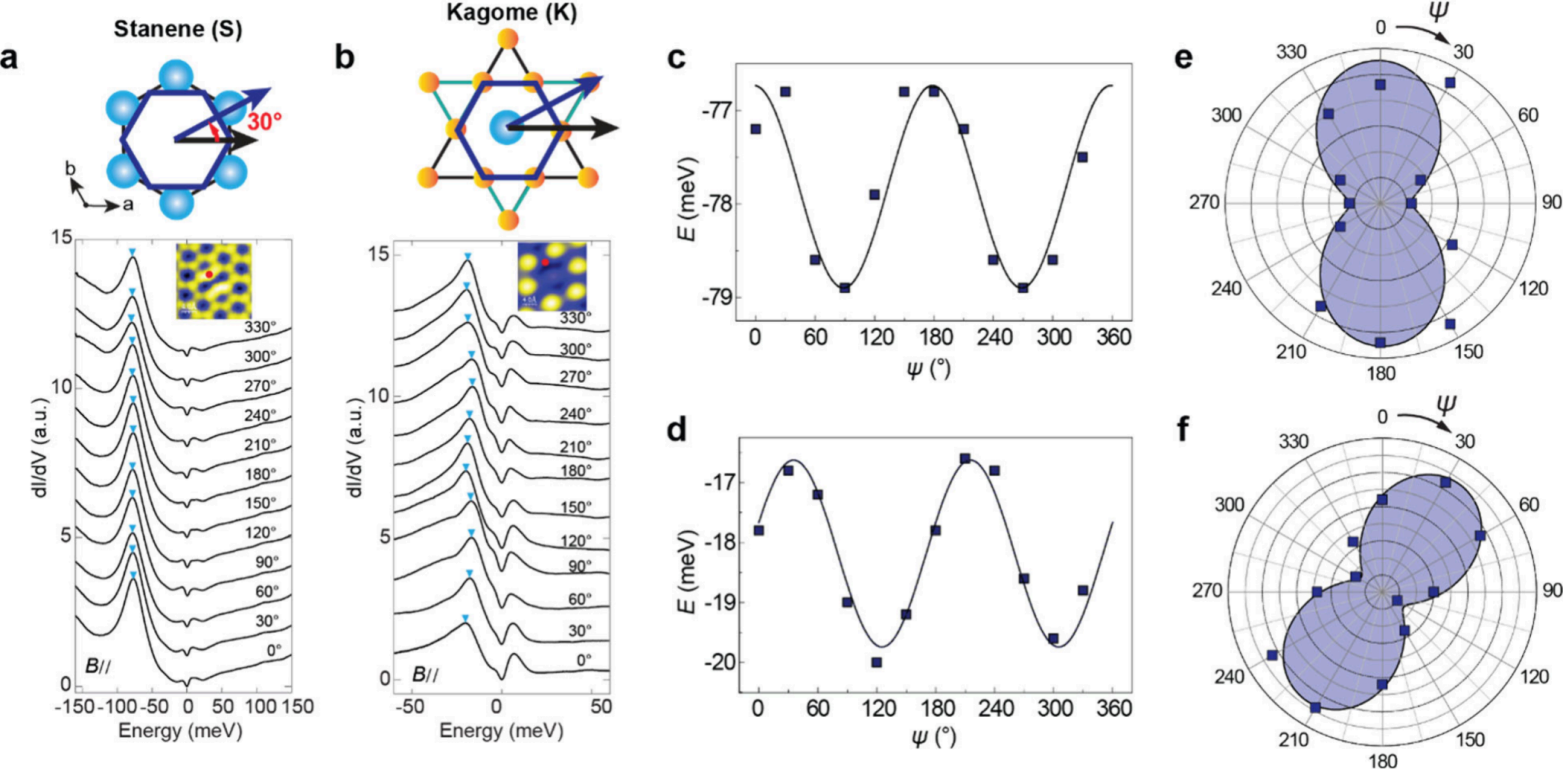
Two-fold oscillating behavior of the defect bound states under an in-plane magnetic field. **a**, d*I*/d*V* spectra taken under in-plane magnetic fields (*B*_∥_) at the red dot of the inset STM image. The magnitude of *B*_∥_ is 1 T, and its direction is denoted by an azimuth angle ψ. Inset: STM image of a Sn divacancy on the Sn layer, set point: *V* = −0.2 V, *I* = 4.0 nA. **b**, d*I*/d*V* spectra taken under in-plane magnetic fields *B*_∥_ of 2 T at the red dot of the inset STM image. The field direction is denoted by its azimuth angle ψ. Inset: STM image of a Sn vacancy in the Kagome layer, set point: *V* = 0.2 V, *I* = 5.0 nA. **c**, The energy positions of the bound state marked by cyan arrows in (**a**) show an oscillatory behavior as a function of ψ. The black fitting curve is *E* = −77.82 + 1.08*sin(2(ψ + 46.53)). **d**, The energy positions of the bound states marked by cyan arrows in (**b**) show an oscillatory behavior as a function of ψ. The black fitting curve is *E* = −18.18 + 1.56*sin(2(ψ + 9.67)). **e**, **f**, Polar plots of the angle-dependent bound states on the Sn and Kagome layers, respectively. A relative rotation ψ = 29.2° is observed between the Sn and Kagome layers, consistent with the 30° rotation between the hexagon unit on the Sn and Kagome layer, as schematically shown in the upper panels of **a** and **b**.

Furthermore, we measured the shift of the defect bound state energy as a function of the azimuth angle ψ with an in-plane magnetic field *B*_∥_ of 1 T (Figures 4a,b and Figure S10). The peak energy positions of the defect bound states as a function of ψ are shown in Figures 4c,d, which can be well fit by a sine function. The oscillatory behavior shows a 2-fold symmetry in both the Sn and Kagome layers, as clearly demonstrated in the angular polar plots in Figures 4e,f. A rotation angle of 29.2° is seen between the spatial anisotropy on the Sn (Figure 4e) and Kagome layers (Figure 4f), consistent with the 30° rotation between their hexagon units (upper panels of Figures 4a,b).

The anisotropic response of defect-bound states to the magnetic field may be attributed to a Stoner–Wohlfarth reorientation of the defect states under the influence of the external field^44^ (see detailed discussion in Supplementary Note 3). However, this mechanism does not explain the anisotropic energy-dependent QPI behavior shown in Figure 2. A more likely scenario is that in the presence of strong spin–orbit coupling, the charge ordering in Kagome materials is strongly coupled to a magnetic field. For example, in ferromagnetic Fe_3_Sn_2_, surface states and QPI patterns have shown a similar 2-fold anisotropy under an in-plane magnetic field.^18^ In antiferromagnetic FeSn^41^ and FeGe,^30,31^ strong magnetic field tunable stripe order and charge density waves have been reported. Even in nonmagnetic AV_3_Sb_5_ (A = K, Rb, Cs) Kagome materials, in-plane field tunable superconductivity, charge density waves, and a magneto-optical Kerr effect were also observed. Specifically, thin-flake RbV_3_Sb_5_ exhibits a 2-fold symmetric superconductivity under an in-plane magnetic field,^45^ and the Kagome metal CsV_3_Sb_5_ presents a 2-fold rotational symmetrical *c*-axis resistivity in both the superconducting and normal states.^46^ Recent magneto-optical Kerr effect measurements of AV_3_Sb_5_ further reveal three-state nematicity, suggesting time-reversal symmetry-breaking.^47^ Moreover, in the strongly correlated Sr_3_Ru_2_O_7_, a compass-like manipulation of electronic nematicity by in-plane magnetic fields has also been observed.^43^ In all cases, strong spin–orbit coupling underpins the entanglement of charge orders and magnetic field, leading to various anisotropic responses to the in-plane magnetic field.

In summary, thin films of antiferromagnetic Kagome FeSn were grown by MBE, and the scanning tunneling differential conductance maps revealed anisotropic QPI patterns near Sn vacancy defects on the Kagome layer. These Sn vacancy defects induced bound states with anomalous Zeeman shifts under an out-of-plane magnetic field. When an in-plane magnetic field was applied, the shift of the bound state energy further exhibited a 2-fold oscillating behavior as a function of the azimuth angle. These findings demonstrate the feasibility of defect engineering to control electronic states in Kagome antiferromagnets for potential applications in nanoscale spintronic devices.

## Methods

### Sample Preparation

The FeSn/SrTO_3_(111) films were prepared by MBE following recipes published elsewhere.^41,42^ The SrTiO_3_(STO)(111) substrates (Nb-doped 0.05 wt %) were first degassed at 600 °C for 3 h followed by annealing at 950 °C for 1 h. During the MBE growth, high-purity Fe (99.995%) and Sn (99.9999%) were evaporated from Knudson cells on the STO(111) substrate with temperatures between 480 and 530 °C.

### LT-STM/S Characterization

STM/S experiments were conducted in a Unisoku ultrahigh-vacuum LT-STM system interconnected to an MBE chamber. All STM/S results were measured at *T* = 4.5 K. A polycrystalline PtIr tip was used, which was tested on Ag/Si(111) films before the STM/S measurements. d*I*/d*V* tunneling spectra were acquired using standard lock-in technique with a small bias modulation *V*_mod_ at 732 Hz.

## Supporting Information Available

Supplementary Notes 1–3: First-principles DFT calculations; calculations of the effective *g* factor; Stoner–Wohlfarth reorientation due to external fields; Supplementary Figures S1–S8: FeSn films grown by MBE on the SrTiO_3_ (STO) (111) substrate; three types of defects in FeSn/STO(111) films; bias-dependent STM imaging of Sn divacancy in the S layer of FeSn/STO(111) films; bias-dependent STM imaging of Sn vacancy on the K layer of FeSn/STO(111) films; simulated STM images of Sn divacancy and Sn vacancy on S and K layers by first-principle DFT calculations; bound states of Sn vacancy on the K layer of FeSn/STO(111) films; topography and d*I*/d*V* maps of Sn bivacancy defect on the S layer of FeSn/STO(111) films; topography and d*I*/d*V* maps of single Sn vacancy defect on the K layer of FeSn/STO(111) films; schematic drawing of anomalous Zeeman shift with electron and hole carriers; field-dependent shift of the defect bound states on the Sn layer (PDF)
