# Supplementary material for: Anisotropic Response of Defect Bound States to the Magnetic Field in Epitaxial FeSn Films
Source: Nano Lett. 2025 Mar 13;25(12):4689–95. doi: 10.1021/acs.nanolett.4c05337 (PMC11951140; doi:10.1021/acs.nanolett.4c05337)
Supplement: Supplementary file 1 — nl4c05337_si_001.pdf [file nl4c05337_si_001.pdf]

Supplementary Information

Anisotropic response of defect bound states to the magnetic field in epitaxial FeSn films

Huimin Zhang[1,2,3*], Zhengfei Wang[4], Michael Weinert[5], and Lian Li[1*]

[1]Department of Physics and Astronomy, West Virginia University, Morgantown, WV 26506, USA

[2]China Key Laboratory of Materials Modification by Laser, Ion and Electron Beams, Dalian University of Technology, Ministry of Education, Dalian, 116024, China

[3]Department of Physics, Dalian University of Technology, Ministry of Education, Dalian 116024, China

[4]Hefei National Research Center for Physical Sciences at the Microscale, CAS Key Laboratory of Strongly-Coupled Quantum Matter Physics, Department of Physics, Hefei National Laboratory, University of Science and Technology of China, Hefei, Anhui 230026, China

[5]Department of Physics, University of Wisconsin, Milwaukee, WI 53201, USA

[*]Correspondence to: huiminzhang@dlut.edu.cn, lian.li@mail.wvu.edu

Note 1. First-principles DFT calculations

Note 2. Calculations of the effective g factor

Note 3. Stoner-Wohlfarth reorientation due to the external fields

Figure S1-S8. Supplementary Figures

Note 1. First-principles DFT calculations

First-principles DFT calculations were carried out in the framework of generalized gradient approximation with the Perdew-Burke-Ernzerhof functionals using the Vienna Ab initio simulation package (VASP). All calculations were performed with a plane-wave cutoff of 500 eV on 7 × 7 × 1 Monkhorst-Pack k-point mesh. In geometric optimization, the atom positions were fully relaxed were until the forces less than 0.02 eV/Å. The Fe_3Sn-terminated (Sn-terminated) surface was modeled by a slab geometry consisting of seven (five) atomic layers with ~15 Å of vacuum, in which the upper three layers were relaxed. The theoretical STM images were simulated using the Tersoff-Hamann approximation with a larger k-points mesh (21 × 21 × 1). The STM tunneling current is proportional to the local density of states of the sample surface at the position of the tip. Therefore, the simulated STM image is the plot of the charge density distribution in a chosen energy window on one horizontal plane above the sample surface. Here, the theoretical energy window is compared to the STM bias voltage, and the vertical position of the horizontal plane is compared to the height of the STM tip. In the simulations, the orbital of the STM tip is considered an isotropic s-wave, and the density is directly obtained from the DFT calculations.

Note 2. Calculations of the effective *g* fator

For Zeeman effect

$$\Delta E = -\mu \cdot B, \qquad (1)$$

where ΔE is the energy shift, μ is the magnetic moment, and B is the applied magnetic field. The slope $\frac{\Delta E}{B}$ is derived from the magnetic-field-dependent plot of the peak position, which is 4.79 ± 0.28 meV·T^{-1} and 2.36 ± 0.12 meV·T^{-1} for the Sn-vacancy defects in the S and K layers, respectively.

Note that the intrinsic value of the magnetic moment of an electron or Bohr magneton is -0.9284×10^{-23} J·T^{-1}. The conventional spin Zeeman effect *g* factor g_s ~2 would lead to 0.058 meV·T^{-1}. Therefore, the 4.79 ± 0.28 meV·T^{-1} and 2.36 ± 0.12 meV·T^{-1} correspond to an effective *g* factor of 165.2 ± 9.7 and 81.4 ± 4.2 for Sn-vacancy defect in the S and K layer, respectively.

Note 3: Stoner-Wohlfarth reorientation due to the external fields

Although anomalous Zeeman shifts provide a plausible explanation of the data, albeit with large effective g factors, a possible alternative explanation is that the defect states exhibit a Stoner-Wohlfarth reorientation due to the external fields. The moments in bulk FeSn are ferromagnetically aligned in-plane and antiferromagnetically out-of-plane. Thus, the vacancies on the two surfaces are likewise expected to be predominantly in-plane. (Vacancy defects, even in non-magnetic materials, often have unpaired electrons.) The in-plane field dependence of sin 2ψ (or equivalently $(\sin\psi)^2$) shown in Figs. 4**c**&**d**) is the form expected for uniaxial or hexagonal anisotropy, consistent with the spins associated with the defect to be in-plane. Application of a perpendicular magnetic field would cause the spins to align with the field. As a function of the field, the spins would start canting until they are saturated, i.e., aligned with the field. The data in Fig. 3 suggests that fields of ~0.5 T are sufficient to overcome the in-plane anisotropy for both defect levels. For larger fields, the bulk FeSn would also be expected to start to align with the fields, which can explain the high-field behavior seen in Figs. 4**c**&**d**. Within this explanation, if the spins were originally aligned mainly in-plane, then positive and negative fields would be expected to shift in the same direction, as seen in Fig. 3 since the spins of the defect states would be perpendicular to the in-plane moments of the Fe atom in both cases.

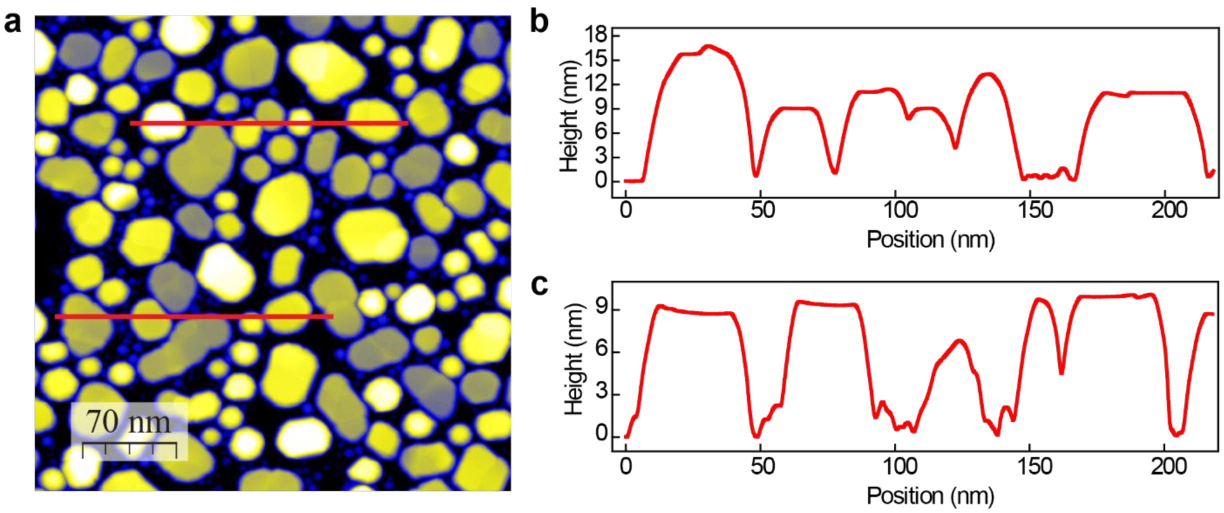

Figure S1. FeSn films grown by MBE on the $SrTiO_3$ (STO) (111) substrate. a, Topographic STM image of epitaxial FeSn/STO(111) films. Setpoint: V = 3.0 V, I = 10 pA. **b**-**c**, Line profiles along the red lines denoted in **a**.

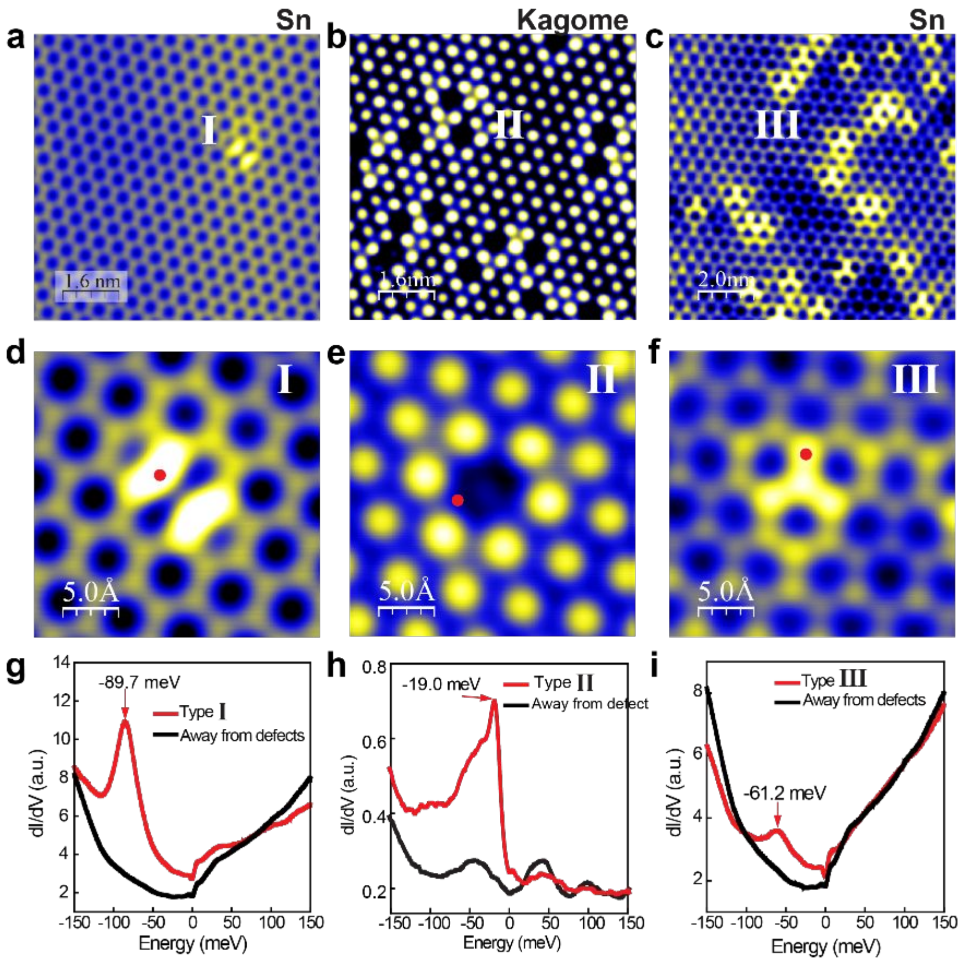

Figure S2. Three types of defects in FeSn/STO(111) films. a-b, Topographic STM image of three kinds of defects marked as Ⅰ, Ⅱ, and Ⅲ. Setpoint: *V* = 0.5 V, *I* = 3.0 nA (**a**), *V* = -0.1 V, *I* = 3.0 nA (**b**) and *V* = -0.5 V, *I* = 3.0 nA (**c**). **c-e**, Atomic resolution images of three types of defects marked as Ⅰ, Ⅱ, and Ⅲ. **f-h**, dI/dV spectra of the defect state (red curve) and away from the defect (black curve).

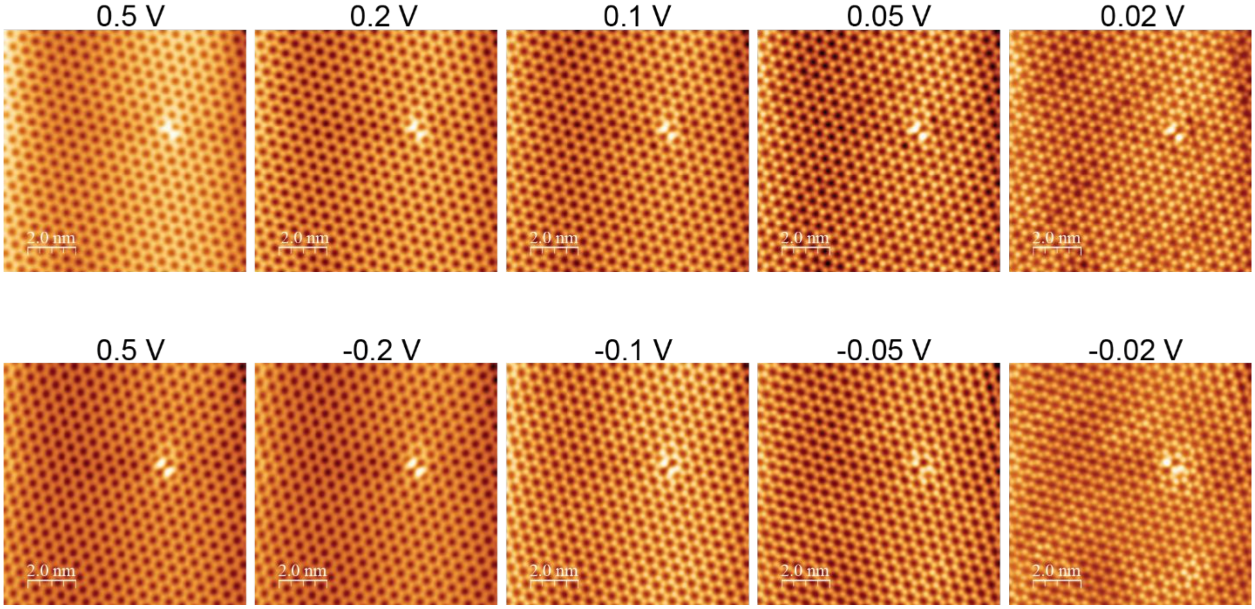

Figure S3. Bias-dependent STM imaging of Sn divacancy in S layer of FeSn/STO(111) films. Setpoint: *I* = 3.0 nA and *V* as specified.

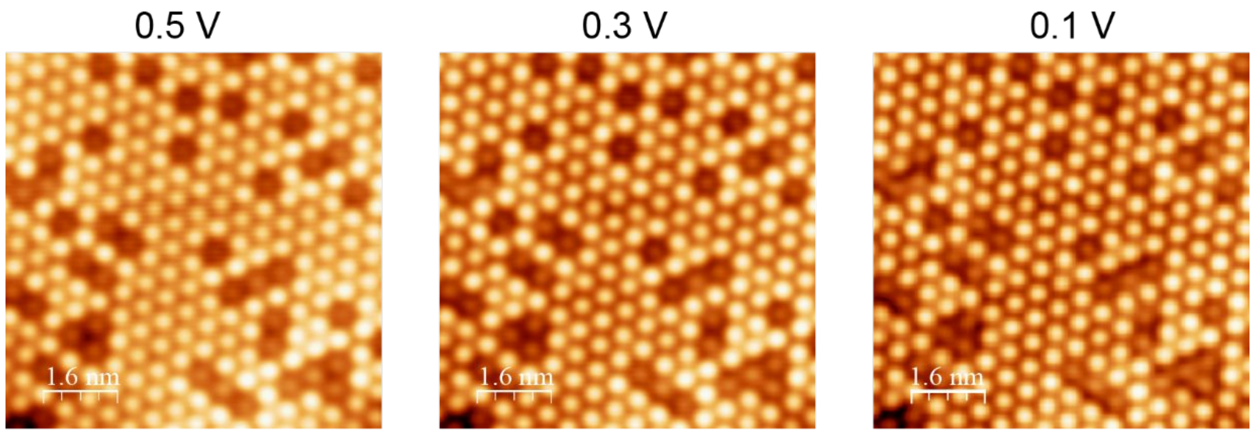

Figure S4. Bias-dependent STM imaging of Sn vacancy in K layer of FeSn/STO(111) films. Setpoint: I = 2.0 nA and V as specified.

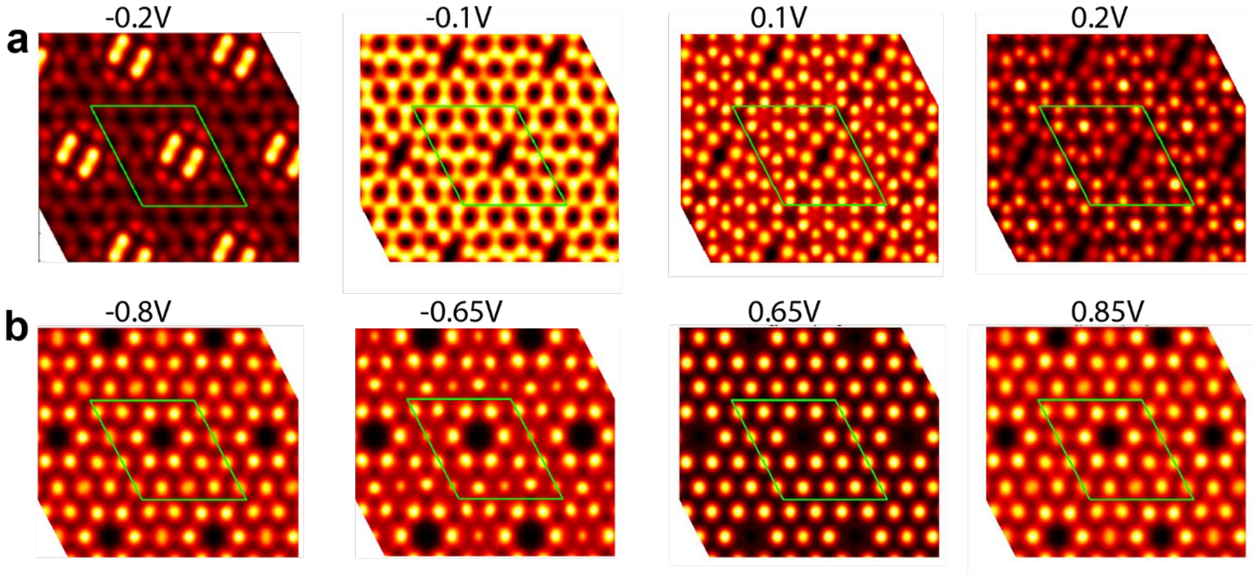

Figure S5. Simulated STM images of Sn divacancy and Sn vacancy in S and K layers by first-principle DFT calculations.

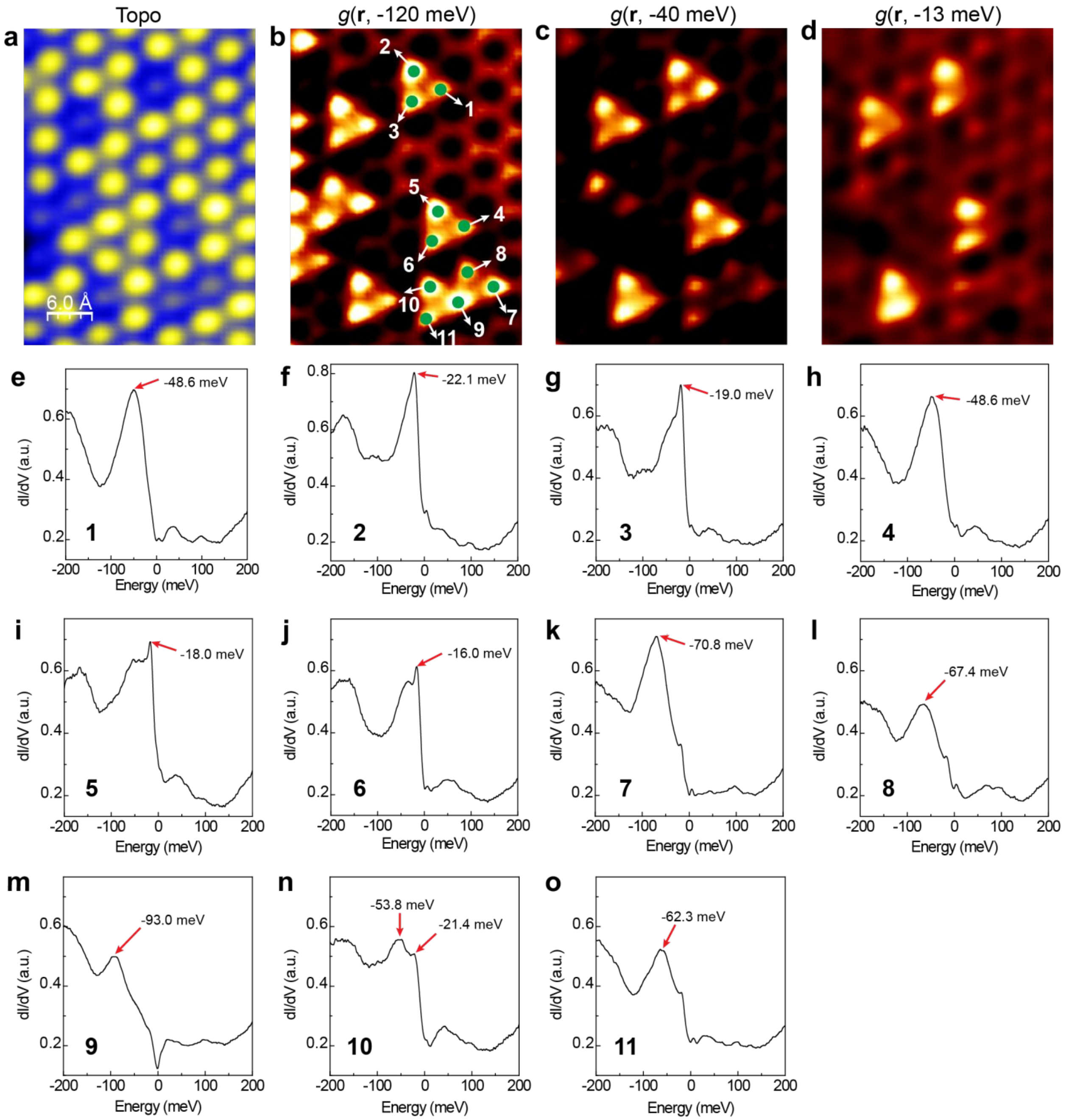

Figure S6. Bound states of Sn vacancy in the K layer of FeSn/STO(111) films. a, Topographic STM image of Sn vacancies in the K layer. Setpoint: V = 0.2 V, I = 3.0 nA. **b**-**d**, dI/dV maps at various energies as labeled. Setpoint: V = 0.2 V, I = 3.0 nA, V_{mod} = 3.0 meV. **e**-**o**, dI/dV spectra revealing the bound states taken at six sites marked in **b**.

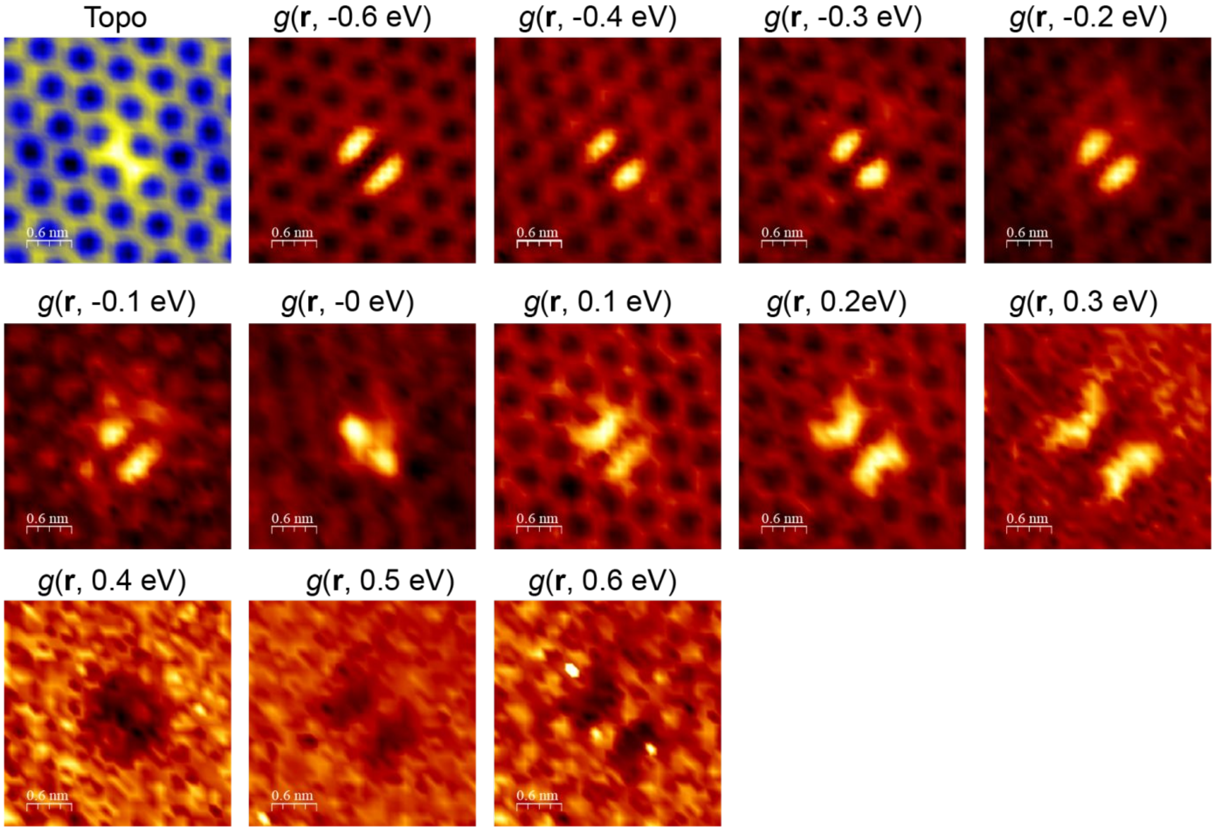

Figure S7. Topography and dI/dV maps of Sn bivacancy defect in S layer of FeSn/STO(111) films. Setpoint: V = 0.6 V, I = 5.0 nA, V_{mod} = 6.0 meV.

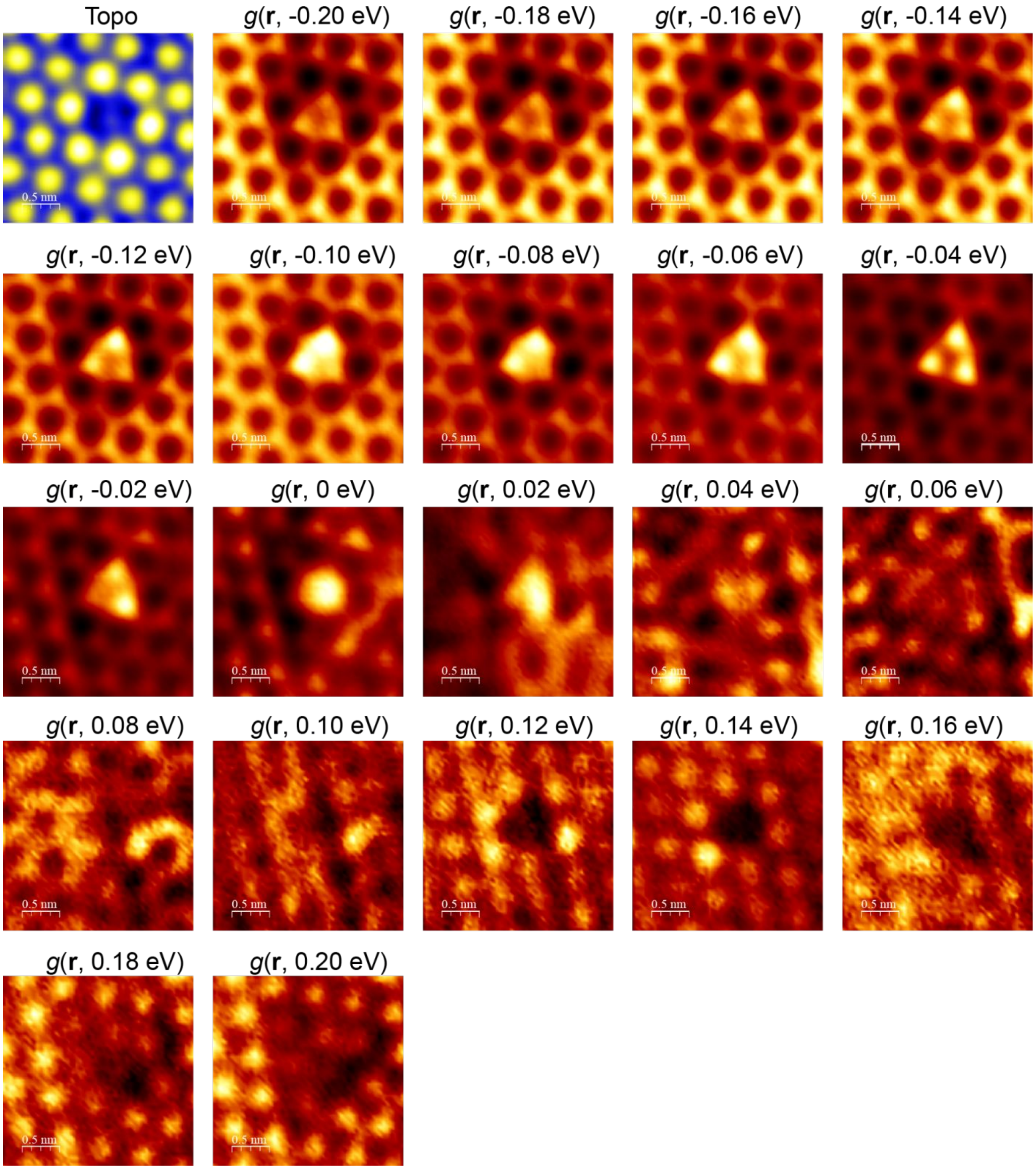

Figure S8. Topography and dI/dV maps of single Sn vacancy defect in K layer of FeSn/STO(111) films. Setpoint: V = 0.2 V, I = 3.0 nA, V_{mod} = 3.0 meV.

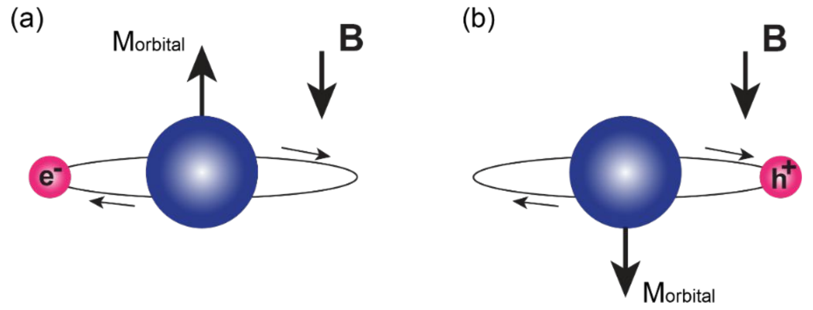

Figure S9. Schematic drawing of anomalous Zeeman shift with electron and hole carriers. (a) The orbital moment $M_{orbital}$ exhibits a diamagnetism with the applied magnetic field B with electron carriers. (b) With hole carriers, the orbital moment $M_{orbital}$ displays a parallel direction with the applied magnetic field B.

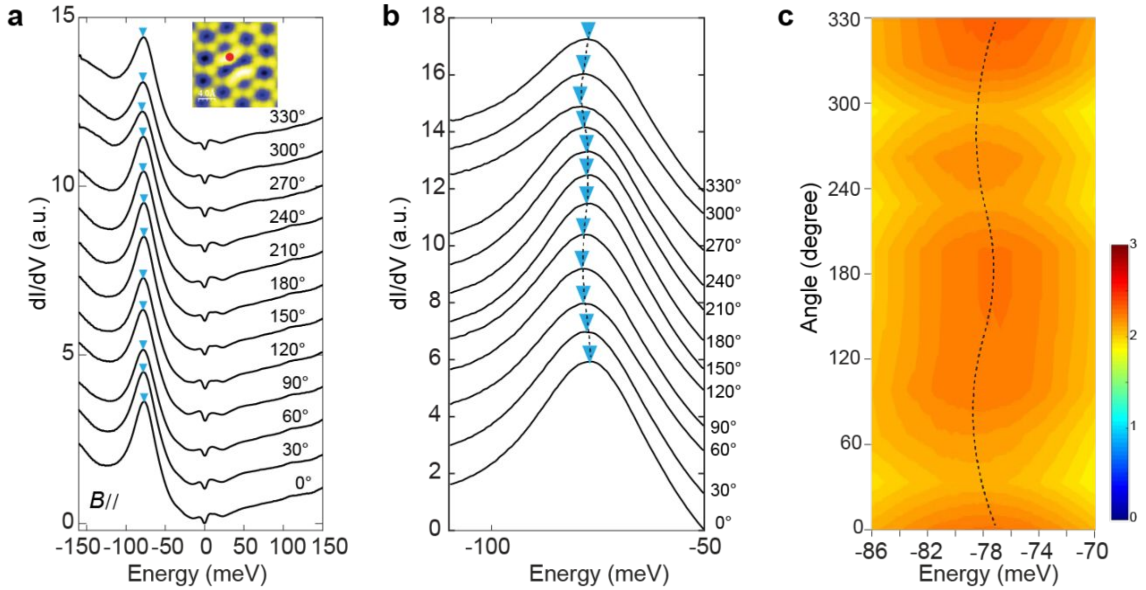

Figure S10. Field-dependent shift of the defect bound states on the Sn layer. a, dI/dV spectra under in-plane magnetic fields ($B_{//}$) taken at the red dot of the inset STM image. The magnitude of $B_{//}$ is 1 T, and its direction is denoted by an azimuth angle ψ. Inset: STM image of a Sn divacancy on the S layer, setpoint: V = -0.2 V, I = 4.0 nA. The peak position is marked by cyan arrows. **b**, Zoom-in of the dI/dV spectra within the energy range [-110, -50] meV. **c**, Color-map of the peak position marked by cyan arrows in **a** & **b**. The dashed line marks the peak position.
